# Supplementary figures and images for: Taxonomic status of Coryphophylax maximiliani Fitzinger in: Steindachner, 1867 with notes on Coryphophylax subcristatus (Blyth, “1860” 1861)
Source: PeerJ. 2025 Sep 19;13:e19841. doi: 10.7717/peerj.19841 (PMC12452941; doi:10.7717/peerj.19841)

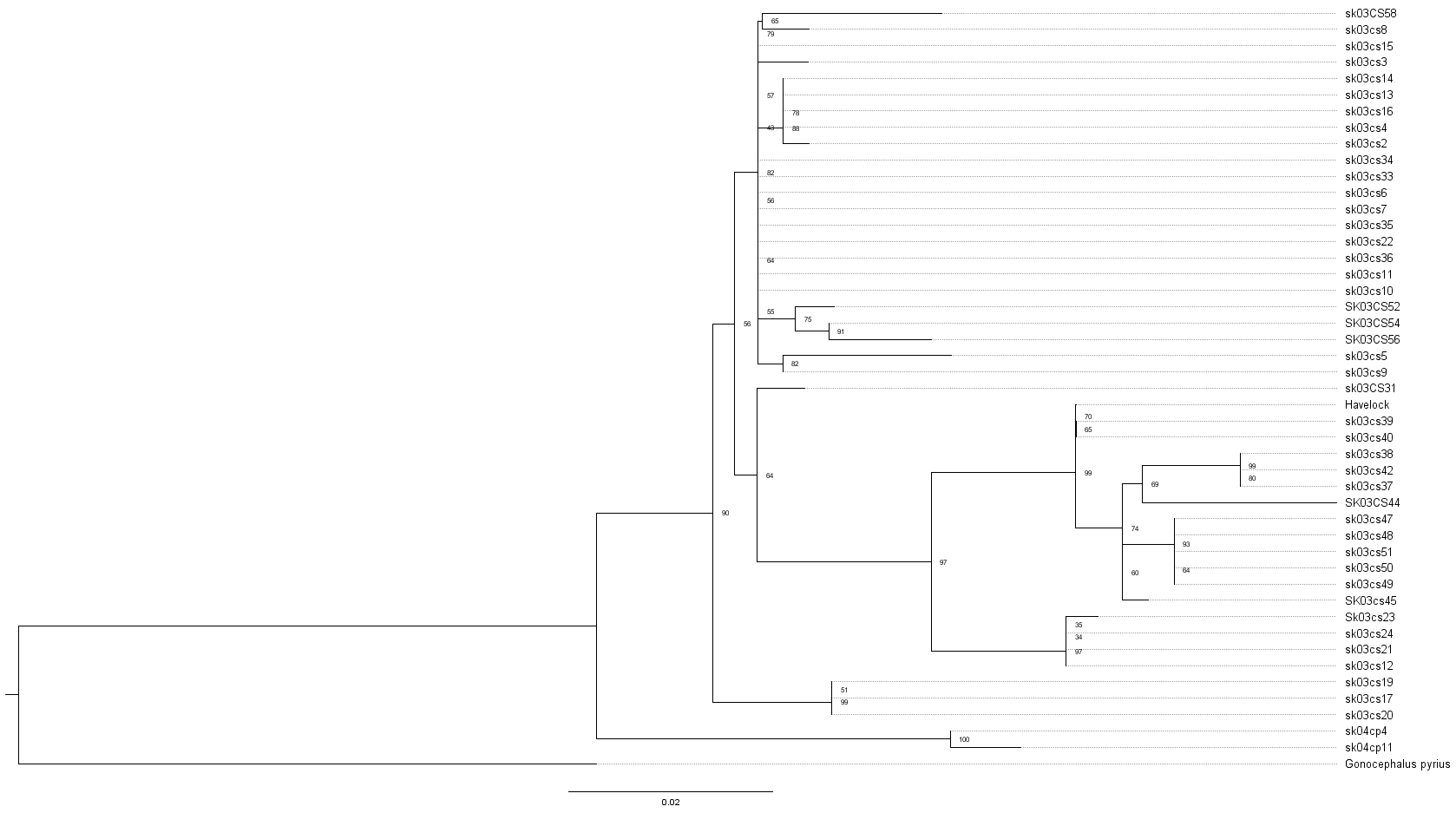

Supplement: Supplemental Information 1 [file peerj-13-19841-s001.png]

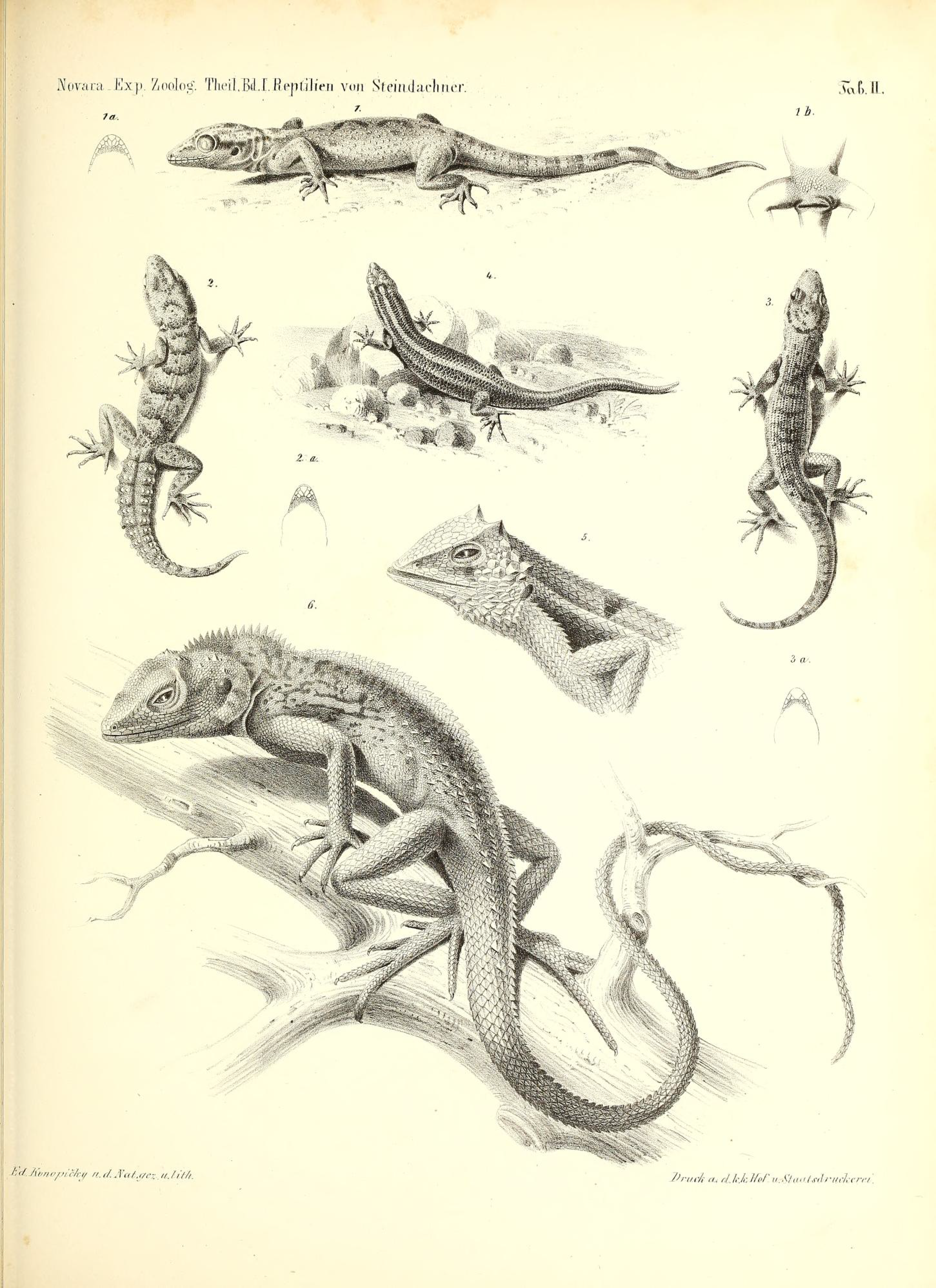

Supplement: Supplemental Information 2 [file peerj-13-19841-s002.tif]
